# Landsat Time Series Reconstruction Using a Closed-Form Continuous Neural Network in the Canadian Prairies Region †

**DOI:** 10.3390/s25051622

**Published:** 2025-03-06

**Authors:** Masoud Babadi Ataabadi, Darren Pouliot, Dongmei Chen, Temitope Seun Oluwadare

**Affiliations:** 1 Laboratory of Geographic Information and Spatial Analysis, Department of Geography and Planning, Queen’s University, Kingston, ON K7L 3N6, Canada; 22mba1@queensu.ca (M.B.A.); oluwadare.t@queensu.ca (T.S.O.); 2 Landscape Science and Technology Division, Environment and Climate Change Canada, Ottawa, ON K1A0H3, Canada; darren.pouliot@ec.gc.ca

**Keywords:** remote sensing (RS), deep learning, Landsat time series reconstruction, closed-form continuous neural network (CFC), Canadian Prairies

## Abstract

The Landsat archive stands as one of the most critical datasets for studying landscape change, offering over 50 years of imagery. This invaluable historical record facilitates the monitoring of land cover and land use changes, helping to detect trends in and the dynamics of the Earth’s system. However, the relatively low temporal frequency and irregular clear-sky observations of Landsat data pose significant challenges for multi-temporal analysis. To address these challenges, this research explores the application of a closed-form continuous-depth neural network (CFC) integrated within a recurrent neural network (RNN) called CFC-mmRNN for reconstructing historical Landsat time series in the Canadian Prairies region from 1985 to present. The CFC method was evaluated against the continuous change detection (CCD) method, widely used for Landsat time series reconstruction and change detection. The findings indicate that the CFC method significantly outperforms CCD across all spectral bands, achieving higher accuracy with improvements ranging from 33% to 42% and providing more accurate dense time series reconstructions. The CFC approach excels in handling the irregular and sparse time series characteristic of Landsat data, offering improvements in capturing complex temporal patterns. This study underscores the potential of leveraging advanced deep learning techniques like CFC to enhance the quality of reconstructed satellite imagery, thus supporting a wide range of remote sensing (RS) applications. Furthermore, this work opens up avenues for further optimization and application of CFC in higher-density time series datasets such as MODIS and Sentinel-2, paving the way for improved environmental monitoring and forecasting.

## 1. Introduction

For several reasons, the Landsat archive is widely regarded as one of the most crucial data sources for studying landscape change. Firstly, the Landsat program has the longest and highest spatial resolution collection of images spanning over 50 years, providing a unique historical record of the Earth’s surface characteristics. This extensive dataset allows for the development of time series algorithms that can monitor and analyze land cover and land use changes over time, enabling the detection of trends and dynamics in the Earth’s system [1,2]. Secondly, Landsat image calibration and processing to surface reflectance is well established, making it a reference point for other satellite systems. Thirdly, the Landsat program’s commitment to free and open access data has revolutionized the field by allowing users to access a vast archive of imagery. These factors, combined with the program’s continuity, advancements in data processing, and its role as a reference instrument, make the Landsat image time series an invaluable resource for understanding and addressing global environmental challenges [3,4]. However, the relatively low temporal frequency and, more importantly irregular, clear-sky observations are some of the main drawbacks of Landsat data and other optical remote sensing (RS) satellites [5,6,7,8].

Reconstructing missing data in optical satellite images is crucial for improving data availability and enabling multi-temporal analysis. For low-resolution earth observation (EO) data with daily or close to daily samples, interpolation or smoothing methods can be used to fill the gaps and generate regularly sampled time series [7]. For example, in 2018, Whitney et al. applied a moving-window polynomial least-squares method to smooth 8-day composite NDVI data from MODIS, minimizing noise and generating daily values, which they used to assess soil salinity in California’s Central Valley, USA [9]. In the case of the Landsat historical data, challenges such as cloud cover, haze, shadows, and sensor artifacts, coupled with the 16-day satellite revisit cycle, result in infrequent and irregularly sampled clear-sky observations. Creating consistent and dense time series data becomes a complex task, particularly when aiming to capture seasonal variations occurring over days to weeks. Conventional interpolation methods often fall short, especially for very sparse and irregular Landsat time series [7,10]. Therefore, further research is necessary to explore the potential of new methods for Landsat time series reconstruction.

Several methods have been used for reconstructing RS irregular time series such as spectral temporal metrics, interpolation, autoregressive (AR) modeling, double logistic modeling, and harmonic modeling [11,12,13,14]. However, most of them are unable to handle and model very sparse Landsat time series effectively. In recent years, there have been numerous research efforts focused on refining the accuracy of Landsat time series modeling by leveraging harmonic models as they have demonstrated superior performance with sparse and irregular Landsat time series data. In one of these studies, Pouliot and Latifovic (2018) developed an imputation-based approach to constrain the harmonic modeling method using AVHRR or climate data (maximum daily temperature and precipitation). Their findings indicated that the utilization of constrained harmonic models enhanced the outcomes of the time series reconstruction, and climate data for imputation yielded superior accuracy compared with the use of AVHRR [7].

Harmonic models have been used for Landsat time series modeling and change detection largely based on the continuous change detection and classification (CCDC) method [5,15]. CCDC has been employed by the United States Geological Survey for development of the Land Change Monitoring, Assessment, and Projection data (LCMAP). Within the framework of CCDC, a harmonic time series model incorporates elements of seasonal variation and trends to estimate surface reflectance. To detect break points and accommodate the diverse spectral responses associated with different types of land cover change, the CCDC algorithm employs a threshold derived from all seven Landsat bands [5]. In follow-up research, the authors introduced a modified version of CCD known as Band-First Probability (CCD-BFP). This approach involves initially assessing the probability of change for each band. Subsequently, these probabilities from each band are aggregated to calculate the overall probability of change [15]. CCDC was designed for time series modeling and change detection where a perfect fit to the observed data is not required as long as the unique temporal–spectral properties are captured for change comparison. Thus, in many cases, the model predictions do not fit the time series well, especially when the signal for a given band cannot be represented by a few harmonic frequencies. For instance, models based on CCDC encounter challenges in accurately modeling time series data of cropped land and identifying their breakpoints. This is primarily because the surface reflectance of cropped areas often exhibits significant variability and unpredictability due to anthropogenic modification [15]. As a result, more suitable techniques are needed to model and reconstruct missing observations in these kinds of challenging Landsat time series. Moreover, improved reconstruction of Landsat time series can enhance temporal information required for numerous applications related to monitoring of snow/ice, water, or vegetation.

The development of deep learning-based approaches in recent years has shown significant potential for improving many RS applications including time series modeling [16]. However, there are still research gaps and limitations in using deep learning methods, as reported in the literature. While deep learning methods have excelled in data modeling, they often struggle with irregular time series datasets and capturing the temporal irregularities present in incomplete time series [16,17]. In other words, most deep learning methods for sequence modeling, such as the recurrent neural network (RNN) or its advanced version the long short-term memory (LSTM) network, are generally designed for regular (evenly spaced) time series modeling and are unable to handle irregular time series observations. In RS, in particular, there has been little research focusing on irregular time series modeling using deep learning techniques.

In 2022, Zhou et al. used an autoencoder-LSTM for similar pixel clustering and a forward–backward LSTM to reconstruct missing data in Landsat time series. To deal with the issue of using irregular time series, they introduced masking layers. In addition, rather than employing conventional LSTM networks, they utilized a forward–backward fusion LSTM network [16]. However, they only utilized a one-year Landsat time series for constructing their model and evaluating its performance, which is insufficient to determine its suitability for reconstructing historical Landsat time series data. In another study addressing irregular time series in RS, Zhang et al. (2024) presented a model called Classifying Raw Irregular Time series (CRIT) for Landsat land cover classification. To this end, they used a masking layer (to mask missed samples) and a transformer architecture for time series classification [18]. Despite the limited research on irregular time series modeling using deep learning models in RS, existing approaches have focused on applications such as land cover classification using irregular time series rather than time series reconstruction or tried to just fill the missing samples at coarse time steps (e.g., 16 days) rather than reconstructing dense regular time series.

Due to the limited studies addressing irregular time series reconstruction in RS, methods used for this purpose in other fields can provide valuable insights into the most promising approaches for evaluation. Baytas et al. introduced a novel version of LSTM termed Time-Aware LSTM (T-LSTM) in 2017, designed to manage irregular time intervals within healthcare patient records. They employed the T-LSTM within an autoencoder framework to classify patients into clinical subtypes. T-LSTM takes irregular elapsed time into account by transforming it into a weight using a time decay function [19]. Nevertheless, clinical data differ significantly from RS time series data due to the inherent uncertainties, noise, and complexities involved in RS data analysis. In another study, Chen et al. (2019) generalized the concept of RNNs to incorporate continuous-time hidden dynamics specified by ordinary differential equations (ODEs), introducing a model known as ODE-RNNs. Continuous neural network structures constructed using ODEs are valuable for modeling data exhibiting complex dynamics. They additionally suggested employing an ODE-RNN within an encoder–decoder framework, creating a sequence-to-sequence model entirely based on ODEs. In such an architecture, a sequence of varying lengths is transformed into a fixed-dimensional representation by the encoder and then reconstructed into another variable-length sequence by the decoder. They showed that their models (an autoregressive ODE-RNN model and encoder–decoder model based on ODE-RNN) outperformed other models such as RNN-decay and RNN-based encoder–decoder for classification, interpolation, and extrapolation/forecasting of irregular time series [20]. Nevertheless, training neural networks based on ODEs is time-consuming due to the utilization of sophisticated numerical solvers for differential equations [21]. This issue becomes even more challenging as the complexity of both the data and the task grows. To address the time-consuming issue of ODE-based models, Hasani et al. (2022) introduced a closed-form continuous-depth (CFC) model, which possesses the modeling capabilities of ODE-based models but eliminates the need for any solver to model data, making it much faster than their ODE-based counterparts. They demonstrated that CFCs exhibit superior performance and less computational burden compared to other baseline models capable of modeling irregular time series, such as Phased-LSTM, RNN-Decay, Bi-directional RNN, and ODE-LSTM, in tasks such as per time-step classification and per time-step regression [22].

**Figure 1:**
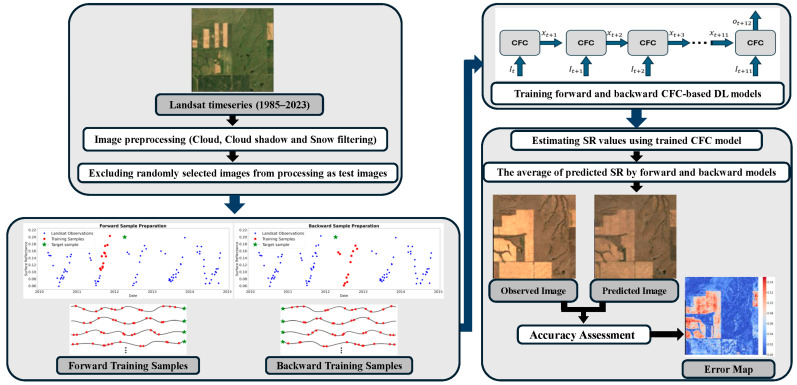
The flowchart of the method used in this study for Landsat time series reconstruction.

This research evaluated CFC as an advanced deep learning method capable of handling irregular and sparse time series for reconstructing the historical Landsat time series from 1985 to present in a sample area in the Canadian Prairies region. The performance of CFC is also compared with that of the CCD method. Figure 1 presents a detailed flowchart outlining the methodology used in the study for Landsat time series reconstruction.

The rest of this paper is organized as follows. Section 2 details the study area and dataset, provides an overview of CFC neural networks and introduces the evaluation method. Section 3 presents and discusses the results. Finally, Section 4 presents our conclusion.

## 2. Materials and Methods

### 2.1. Study Area and Dataset

**Figure 2:**
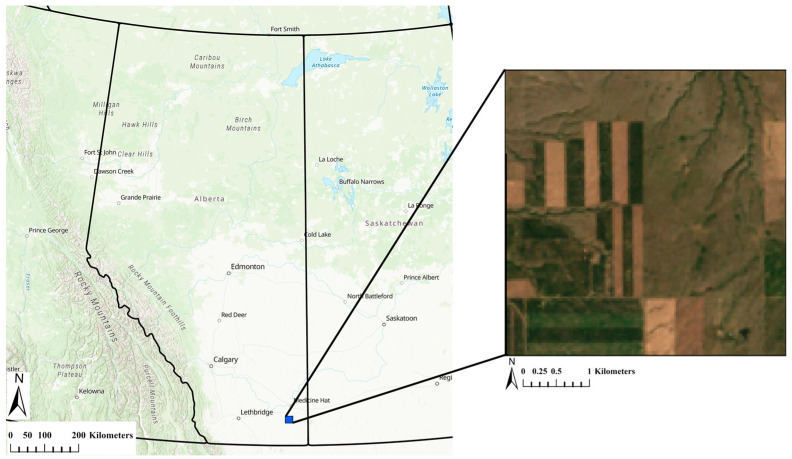
The study area situated in southeast Alberta. The right section provides an overview of a Landsat 5 TM image captured on 27 July 1999, displayed with a true-color band composition. Basemap: Esri, TomTom, Garmin, FAO, NOAA, USGS, EPA, NRCan, Parks Canada.

The Great Plains in North America, including Canada’s Prairie region, comprises a significant portion of the world’s grasslands, with Canada holding 16% of this landmass. Specifically, the Canadian Prairies, spanning Alberta, Saskatchewan, and Manitoba, host 89% of the nation’s grasslands, both native and cultivated [23]. The study area is part of the Canadian Prairies region located in southeast Alberta, Canada (Figure 2). The main land covers include grasslands and agricultural lands, with an elevation range between 953 and 1083 m above the mean sea level (MSL). This region was selected because the agricultural areas are temporally dynamic due to the seasonal climate and its variability, soil preparation for planting, and harvesting. Grasslands also have unique temporal properties that can help separate native from cultivated land and could benefit from better temporal characterization [24,25,26]. The dataset utilized in this study consists of atmospherically corrected and orthorectified surface reflectance images, generated from the data captured by the TM (Landsat 5), ETM+ (Landsat 7), OLI (Landsat 8), and OLI2 (Landsat 9) sensors. The Landsat data used in the study were obtained via the Google Earth Engine platform. The study primarily focuses on the visible and near-infrared (VNIR) spectral bands, covering a wavelength range of 0.43 to 0.90 μm, as well as the short-wave infrared (SWIR) spectral bands, which extend across a wavelength range of 1.55 to 2.35 μm. These spectral ranges are crucial for analyzing and monitoring changes across the Earth’s surface. The image time series spans 39 years, covering the period from 1985 to 2023.

### 2.2. CFC Neural Networks

Continuous neural network architectures based on ODEs offer powerful models for capturing complex dynamics in data. By transforming the depth of traditional neural networks and the time step in RNNs into a continuous vector field, these models allow for parameter sharing, adaptive computations, and function approximation, making them well-suited for handling irregularly sampled data [20]. These continuous-depth (or continuous-time) models have demonstrated potential in various applications, including modeling sequential and irregularly sampled data [27,28], hyperspectral image classification [29], capturing spatiotemporal dynamics [30], and forecasting [31]. ODE-based neural networks, despite their competitive performance compared to advanced discretized recurrent models, suffer from slow training and inference due to the reliance on complex numerical differential equation (DE) solvers. This issue worsens as data, task, and state space complexity grow [32].

To address this, Hasani et al. introduced a CFC neural network in 2022, which eliminates the need for numerical solvers by leveraging a closed-form solution [22]. These models retain key features of liquid-state neural networks, such as flexibility, causality, robustness, and interpretability, but are significantly faster and more scalable. Unlike traditional ODE-based models that require iterative integration, CFC explicitly models time in its formulation, enabling efficient processing of irregular time steps.

The key advantage of CFC is its explicit time dependence, which allows the network to incorporate time intervals directly into its computations. This feature ensures that the model dynamically adjusts to varying time gaps between observations, making it particularly effective for handling non-uniformly sampled data. CFC also employs a time-gated mechanism, which regulates memory retention based on the elapsed time between observations. The sigmoidal time gates adjust the influence of past states, preventing rapid memory decay while ensuring smooth adaptation to irregular sampling intervals.

**Figure 3:**
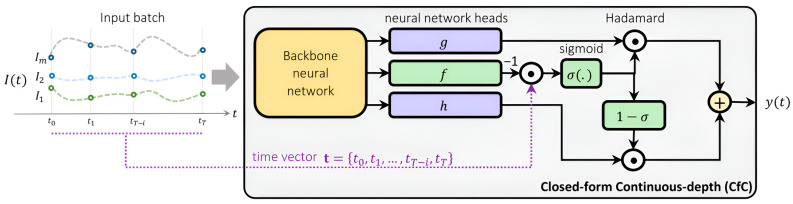
The architecture of the CFC neural network. A backbone neural network layer processes the input signals and distributes them to three head networks: g, f, and h. In this configuration, f serves as a liquid time constant that regulates the sigmoidal time gates, while g and h create the nonlinear components of the complete CFC network [22].

The CFC neural architecture is provided in Figure 3. Rather than training the three neural network instances f, g, and m individually, they are designed to share the initial layers through a backbone structure. This backbone neural network layer processes input signals and channels them into the head networks g, f, and m. Here, f functions as a liquid time constant for the network’s sigmoidal time gates, directly incorporating time information, while g and m contribute to the nonlinear aspects of the overall CFC network. Consequently, this shared architecture enables the model to learn common representations, enhancing the speed and stability of the learning process [22,31].

The hidden states, ${x(t)}^{D\times 1}$ with D hidden units at each time step t, can be explicitly calculated using Equation (1):

(1) $$x(t)=\overset{\mathrm{Time}\text{-}\mathrm{continuous}\;\mathrm{gating}}{\overbrace{\sigma \left(-f\left(x,I;\theta_{f}\right)t\right)}}⊙g\left(x,I;\theta_{g}\right)+\overset{\mathrm{Time}\text{-}\mathrm{continuous}\;\mathrm{gating}}{\overbrace{\left[1-\sigma \left(-\left[f\left(x,I;\theta_{f}\right)\right]t\right)\right]}}⊙m\left(x,I;\theta_{h}\right)$$

where I(t) denotes the system input and t represents the time step. The functions f, g, and h are neural networks characterized by parameters $\theta_{f}$, $\theta_{g}$ and $\theta_{h}$, respectively. Here, σ denotes the sigmoid function, and ⊙ indicates the Hadamard (elementwise) product.

### 2.3. CFC Time Series Modeling

The CFC implementation used in this study for reconstructing the Landsat time series involved multiple steps. First, snow, cloud, and shadow observations were filtered out from the historical Landsat image time series. Next, training samples were extracted from the filtered series and prepared to train the CFC-based model. Finally, the trained model was used to reconstruct missing Landsat data, and the model’s performance for time series reconstruction was evaluated. Each step is detailed in the following sections.

#### 2.3.1. Contaminated Observation Filtering

For Landsat image preprocessing, an important step in this study was to remove clouds, cloud shadows, and snow. This was performed using a two-step method described in [5]. To this end, pixels were initially masked using the Fmask object-based algorithm [33]. While the Fmask algorithm offers fairly precise masks for identifying clouds, cloud shadows, and snow, it still has limitations. Additionally, it may struggle to distinguish other temporary changes like dense aerosols, smoke, or flooding, which could be mistaken for changes in land cover. Consequently, to identify outliers that were not initially detected by the Fmask algorithm, an additional step called Tmask was implemented. This step involves initially estimating a time series model using the observations and harmonic time series modeling, followed by identifying outliers through a comparison between the model estimates and Landsat observations [5].

#### 2.3.2. Training Sample

**Figure 4:**
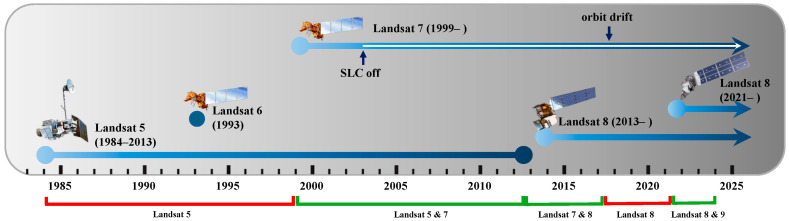
The Landsat missions’ timeline from 1985 to the present.

From 1985 to 2024, considering Landsat 5, 7, 8, and 9 satellites, there were periods when one or two Landsat satellites were actively capturing images of the Earth’s surface; these periods are shown in Figure 4 with red and green markers, respectively. In reference to Landsat 7, the small white arrow within its timeline arrow in Figure 4 shows the period when data were collected without the Scan Line Corrector (SLC). Landsat 7’s SLC, responsible for compensating for the satellite’s forward motion, failed on 31 May 2003. Consequently, in this SLC-off mode, data exhibit zig-zag pattern gaps, although the ETM+ still captures around 78 percent of the data for each scene [34,35]. Furthermore, although Landsat 7 remains in orbit and continues to collect surface data, we only used its data up to August 2017. After that point, the satellite’s orbit began drifting outside its nominal mission parameters, shifting toward earlier acquisition times due to limited onboard fuel. This shift makes Landsat 7 data less reliable for scientific studies requiring precise quantitative analysis [35]. Given the critical importance of data consistency in time series analysis, we excluded any Landsat 7 data acquired after August 2017.

Accurate training and test samples are essential for developing deep learning models. For this, we set aside 15% of images with at least 60% clear observations for each season as test images. Selecting test images with a minimum of 60% clear observations ensured high-quality samples for assessment, as images with extensive cloud, shadow, or snow cover often suffer from issues like haze and cloud adjacency effects. This process yielded 116 test images, with the distribution across seasons shown in Table 1.

**Figure 5:**
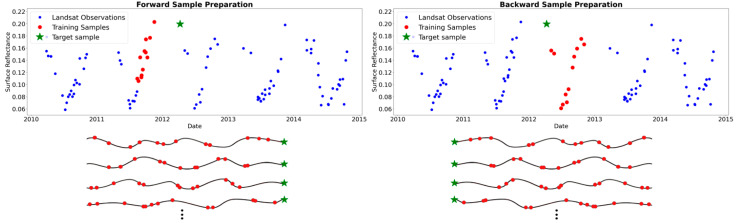
Training sample preparation in forward (**left**) and backward (**right**) approaches.

Training samples were used for forward and backward model training. In the forward case, for predicting each target sample, its 12 previous clear observations were used as training data to predict the target sample. In the backward case, a similar setting was used, but instead of the previous clear observations, the next 12 clear observations following the target observation were used as training data (Figure 5). The selection of 12 as the number of training observations was made during the parameter-setting process and through trial-and-error experimentation. Sample data preparation using available clear-sky observations resulted in approximately 10,000,000 forward samples and another 10,000,000 backward samples. In both cases, 90% of the data was allocated for training, while the remaining 10% was used as a validation set to monitor model performance across epochs and to mitigate overfitting or underfitting.

#### 2.3.3. CFC Implementation

**Figure 6:**
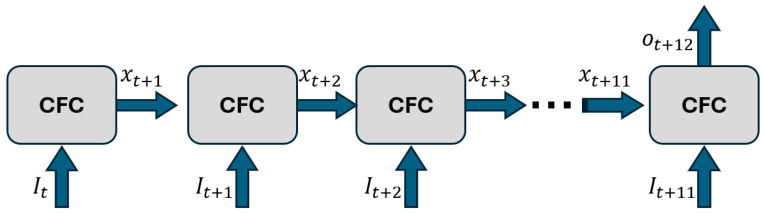
The architecture of a CFC deep neural network.

CFC is designed around forecasting and neural ODEs are, in theory, better for extrapolation than other deep learning approaches [20], which is an important advantage for many applications. Various versions of CFC models exist. In this research, we employed a CFC model integrated within a mixed memory architecture, where the CFC establishes the memory state of an RNN—LSTM in this case to avoid vanishing gradients. This variant is referred to as CFC-mmRNN (described in [22]). The integration of CFC with an RNN architecture results in a deep learning model that the CFC is wrapped inside a recurrent neural network, as depicted in Figure 6, enabling the efficient capture of long-term dependencies in irregularly sampled time series. In this figure, I(t) indicates the input, x(t) represents the hidden state computed by CFC, and o(t) shows the output value of the model at time t. By leveraging explicit time dependence and a time-gated mechanism, the CFC-mmRNN model dynamically adjusts to variable time gaps, ensuring robust handling of irregular satellite time series data.

The CFC-mmRNN model employed in this study comprises 128 recurrent units and three backbone layers, each containing 128 hidden units with “relu” as the backbone activation function. This configuration resulted in a total of 202,374 trainable parameters. The network parameters were optimized using the Adaptive Moment Estimation (Adam) optimizer [36]. The initial learning rate was set to 0.001 and was reduced progressively using a decay rate of 0.98 to enhance convergence. A dropout rate of 0.1 was applied to the backbone layers to mitigate overfitting by randomly deactivating neurons during training. The input batch size was set to 8192, and the total number of epochs was set to 150, which resulted in 178,200 iterations. All training and inference processes performed in this study were conducted using Python 3.11 and TensorFlow 2.15 on an NVIDIA T1000 GPU (Santa Clara, CA, USA).

#### 2.3.4. Evaluation

To evaluate the accuracy, the root mean squared error (RMSE) was computed with the hold-out images. Results were compiled for the full time series data and for four seasons defined as spring (May–June), summer (July–August), fall (September–November), and winter (December–February). Predicted images, difference images between the reference and predicted, and error histograms, for example, images, were generated for visual evaluation. To investigate the impact of sparsity on time series reconstruction, we randomly removed between 10% and 90% of the clear-sky observations from each season of each time series before training. This created ten different scenarios, including the original density level and nine manipulated levels. For the dropout rate, we computed the average number of clear-sky observations per year to help define the minimum sample requirements that should be used with either method. For comparison, the CCD method (as the reference method) was also included as it has been widely used in numerous RS applications to date. We used the latest version of python lcmap-pyccd code (version 2021.7.19) acquired from the USGS for CCD experiments [37].

## 3. Results and Discussion

### 3.1. Comparison of CCD and CFC for Landsat Image Reconstruction

**Figure 7:**
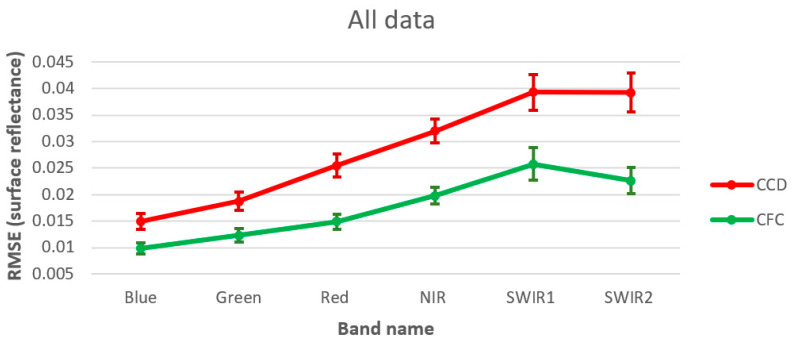
Results of test image reconstruction based on RMSE.

**Figure 8:**
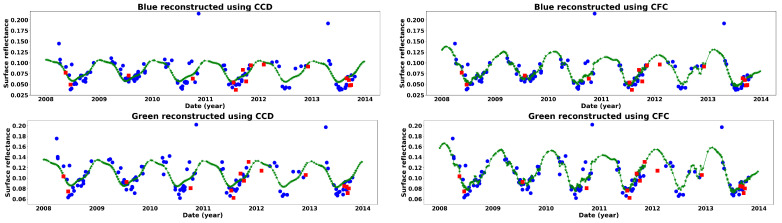

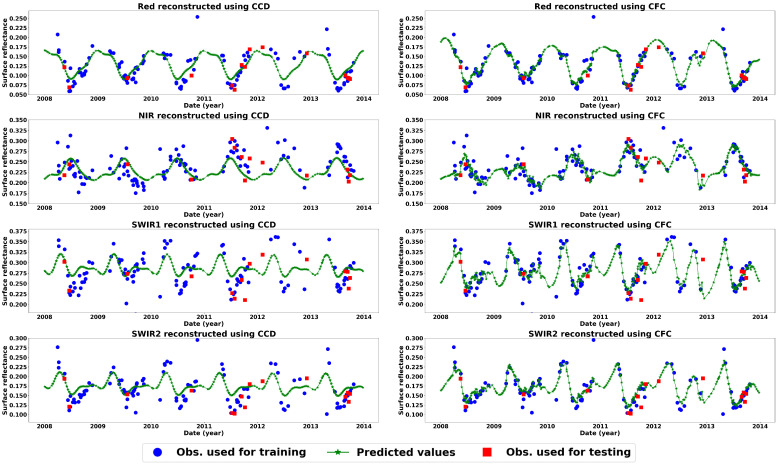
Results of time series reconstruction of a sample grassland pixel in the study area using CCD (**left**) and CFC (**right**) for different bands from 2008 to 2014.

The results of the Landsat time series reconstruction shows that CFC was better able to estimate the hold out test image observations compared to CCD. Table 2 and Figure 7 present the reconstruction results for all test images using both CCD and CFC methods, evaluated based on RMSE. Additionally, Table 2 shows the improvement of CFC over CCD, while Figure 7 illustrates the error confidence levels for each band. As the results indicate, the CFC method achieved higher accuracy across all six bands, with improvements ranging from 33% to 42%. The greatest improvement was observed for the SWIR2 band, while the SWIR1 and blue bands showed the smallest improvement. Figure 7 also reveals that CFC produced narrower confidence intervals compared to CCD across all six bands. Figure 8 illustrates the time series reconstruction results for a sample grassland pixel across different bands over six years from 2008 to 2014. In this figure, observations used for training and testing are presented by blue circles and red squares, respectively. The predicted values are also presented by the green line and stars. Based on Figure 8, the CFC predicted missing values in the time series with significantly higher accuracy than CCD. Notably, the superior performance of CFC over CCD is even more evident in time series with greater complexity, such as the SWIR bands. Here, complexity refers to the general seasonal fluctuations. For the visible bands and the grassland example, there is one clearly defined seasonal cycle, whereas for NIR and SWIR bands there are two cycles with varying amplitudes.

**Figure 9:**
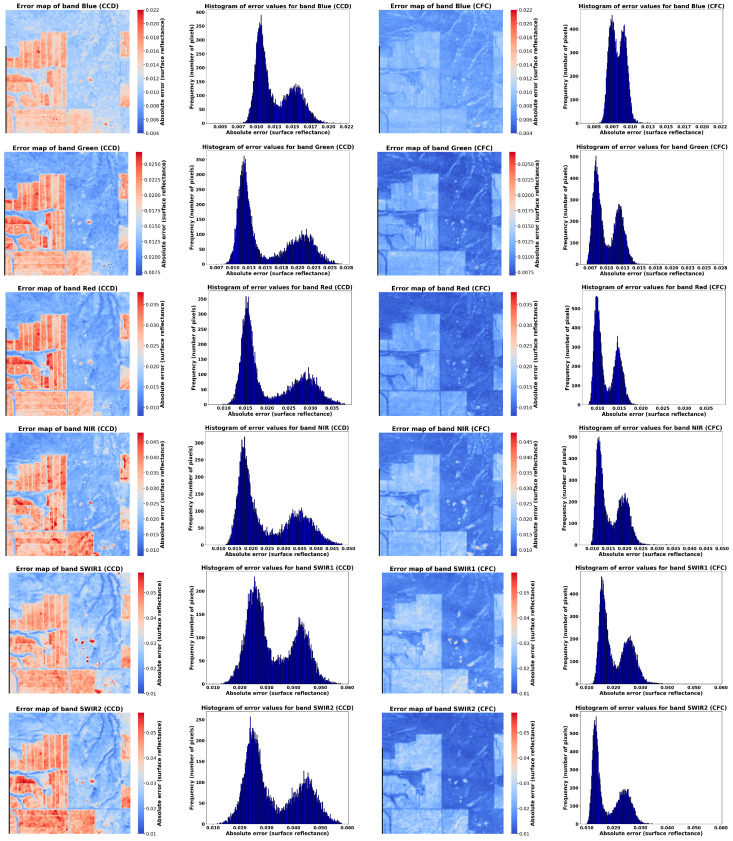
Average of error maps and histogram of error values on average for reconstructed test images using CCD (**left**) and CFC (**right**).

**Figure 10:**
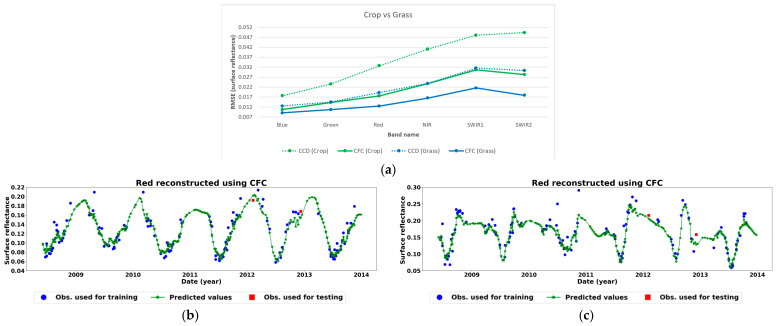
(**a**) Reconstruction errors of test image bands for different land cover types based on RMSE. (**b**) A sample grassland pixel in the red band reconstructed using CFC. (**c**) A sample cropland pixel in the red band reconstructed using CFC.

Figure 9 presents the average error maps and error histograms based on the absolute difference for the 116 test images reconstructed using CCF and CCD methods. The results indicate that CFC consistently outperformed CCD in image reconstruction across all six bands, as demonstrated by both the error maps and histograms. Figure 10 further illustrates the reconstruction errors for each land cover type across different image bands. It is noteworthy that based on the results, errors associated with cropland areas (visible as rectangular regions in the error maps and green lines in Figure 10a) are higher than those for grassland areas. This distinction is also evident in the error histograms, which display two distinct peaks (Figure 9), each corresponding to a specific land cover type. The underlying reason for this difference is the more complex temporal patterns and the higher reflectance in cropland time series compared to grasslands, which often display greater variability and unpredictability (Figure 10b,c). This complexity in cropland dynamics contributes to higher error rates in those areas.

**Figure 11:**
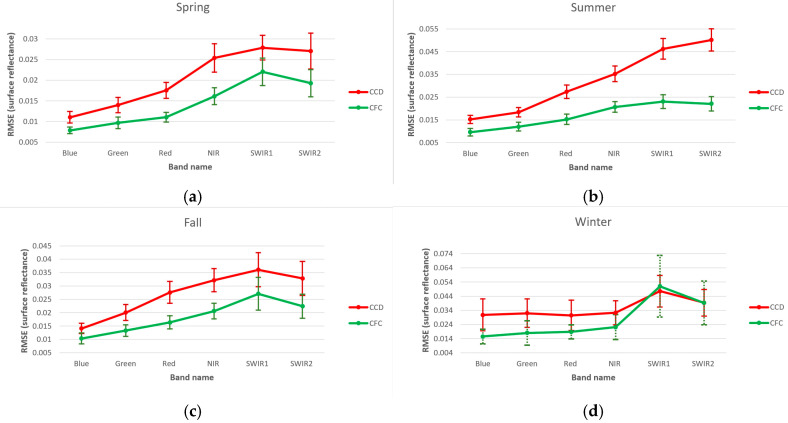
Results of image reconstruction based on RMSE for (**a**) spring, (**b**) summer, (**c**) fall and (**d**) winter.

**Figure 12:**
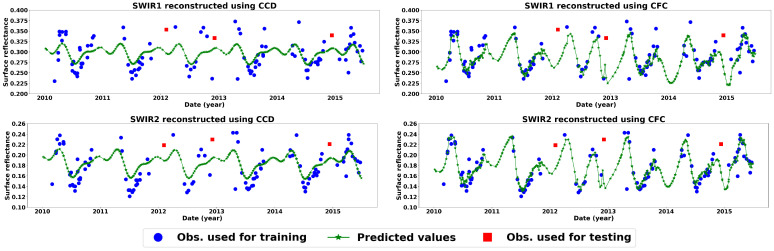
Results of time series reconstructions for a sample grassland pixel in the study area using CCD (**left**) and CFC (**right**) for SWIR bands from 2010 to 2015. Although variations arise due to cloud cover and haze around the winter test samples (red dots), CCD yielded a lower RMSE for these samples, as it is more closely centered around the time series mean.

Figure 11 presents the average of the test image reconstruction results for different seasons and across different bands. The CFC model achieved higher accuracy and confidence levels for all seasons and bands, except for SWIR1 and SWIR2 in winter. This slight advantage of the CCD model in winter for these two bands can be attributed to the reduced density of clear observations during the winter months, leading to a scarcity of training data. Unlike the CFC model, the CCD method employs a harmonic time series model, which incorporates seasonal variations and trends to estimate surface reflectance. This approach allows the CCD model to perform more reliably in cases of very sparse and irregular time series, as it is inherently more constrained than the CFC model under such conditions. Additionally, winter months tend to have increased noise and a higher likelihood of pixel contamination from clouds and haze, impacting the quality of the recorded surface reflectance. This effect is illustrated in Figure 12, which shows an example time series of a grassland pixel for bands SWIR1 and SWIR2 reconstructed using both the CFC and CCD models. In this example, red squares mark three of the test observations in winter and the predicted values are presented by a green line. Another factor contributing to the higher accuracy of the CCD model in these instances is its tendency to remain closer to the mean of the time series relative to CFC. Thus, it is less likely to predict larger residual errors but sacrifices the model fit.

**Figure 13:**
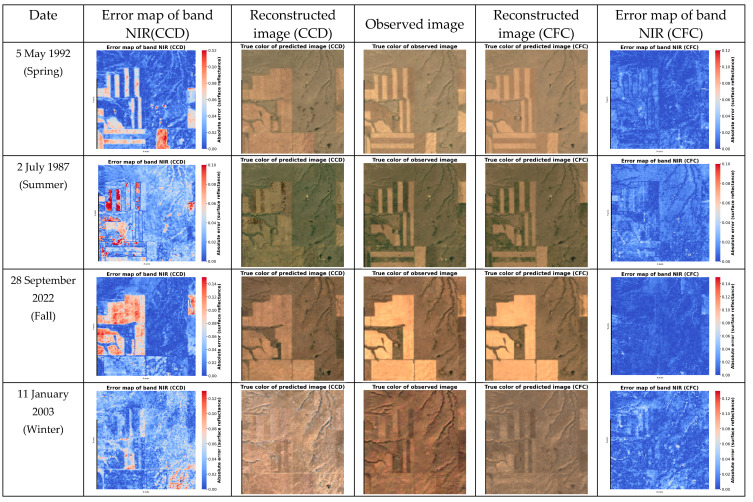
Image reconstruction using CCD and CFC for four test images, each selected from a different season.

Figure 13 presents several examples of test image reconstructions across different seasons using both the CFC and CCD models, along with error maps for the NIR band predictions. The results indicate that the CFC model consistently reconstructed the test images with greater accuracy, closely matching the observed images. In contrast, reconstructions using the CCD model show more significant differences, with the majority of errors related to cropland areas. This difference is due to the higher variability and frequent changes in cropland areas compared to the more stable grassland regions.

### 3.2. Assessing the Effect of Density on Landsat Time Series Reconstruction

**Figure 14:**
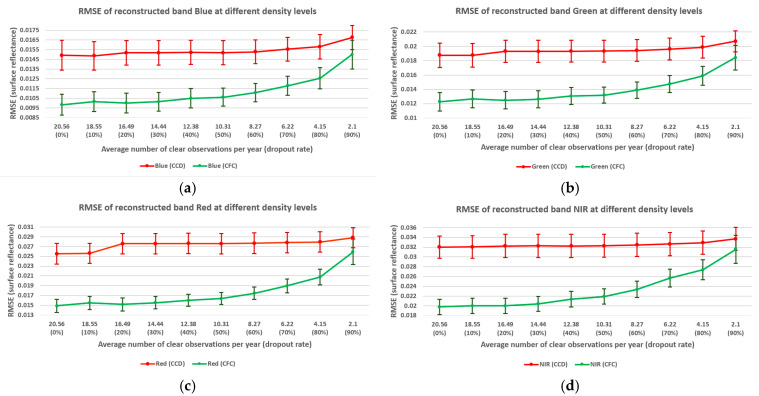

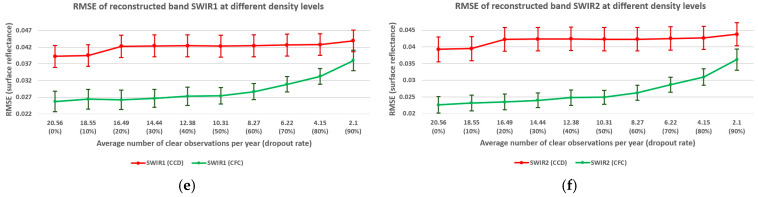
Relation between observation density and RMSE of test image bands reconstruction.

This section analyzes the impact of observation density on Landsat time series reconstruction using the CCD and CFC methods. As outlined in the methodology section, between 10% and 90% of clear observations from each season were randomly selected and excluded from further processing to evaluate how varying density affects time series reconstruction. For each of these dropout rates, the average number of clear observations per year was calculated to indicate where CFC results should not be used or used with caution. Figure 14 illustrates the relationship between observation density and reconstruction error across test image bands. In each plot, the *x*-axis represents the yearly average number of clear observations (density) and the dropout rate, while the *y*-axis indicates the reconstruction error for each test image band, measured by the RMSE. The margins around each point denote confidence interval errors.

The results indicate that as observation density decreases, the CCD method exhibits a smaller increase in error compared to the CFC method, demonstrating that CCD is more resilient to sparsity. However, across all bands and density levels, the CFC method consistently achieved higher overall reconstruction accuracy than CCD. In terms of error confidence, while CFC generally provides narrower confidence intervals compared to CCD, no significant widening of confidence intervals was observed for the methods as observation density decreased.

It is also important to note that the reconstruction accuracy of test image bands using the CFC method drops sharply once approximately 50% of clear observations are excluded, particularly when the density falls below 10 clear observations per year. For the CCD method, there is a slight increase in RMSE for the red, SWIR1, and SWIR2 bands when the density drops to 16 per year. However, beyond this point, the CCD method maintains stable RMSE values across all bands, showing no substantial increase in error even at the lowest density level, where only two observations per year remain on average. This suggests that CCD is notably more robust for very sparse observations and these land cover conditions.

**Figure 15:**
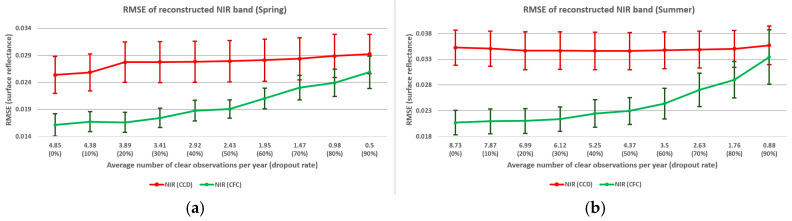

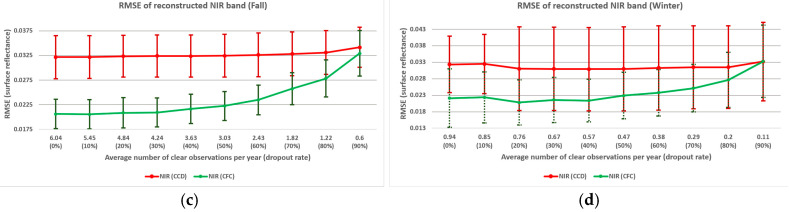
Relation between observation density and RMSE of NIR band reconstruction for (**a**) spring, (**b**) summer, (**c**) fall, and (**d**) winter.

**Figure 16:**
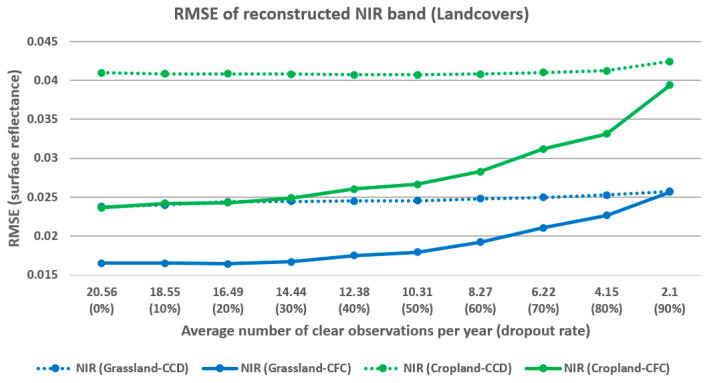
Relation between observation density and RMSE of NIR band reconstruction for different land covers.

The relationship between observation density and RMSE for NIR band reconstruction is further analyzed across different seasons (Figure 15) and land covers (Figure 16). In Figure 15, the *x*-axis represents the yearly average number of clear observations for each season and the dropout rate. Overall, the relationship between density and reconstruction error displays similar patterns across both land covers and seasons. Consistent with previous observations, the CFC method experiences a significant increase in RMSE when 50% of clear observations are excluded. As shown in Figure 16, cropland areas exhibit lower reconstruction accuracy compared to grasslands for both the CCD and CFC methods. Additionally, in cropland areas, the CFC method shows a more pronounced increase in RMSE as density decreases, indicating that cropland reconstruction is more sensitive to sparse observations than grassland reconstruction. However, it is important to note that for cropland (as a more complex land cover), the CFC method provides a greater improvement over the CCD method.

**Figure 17:**
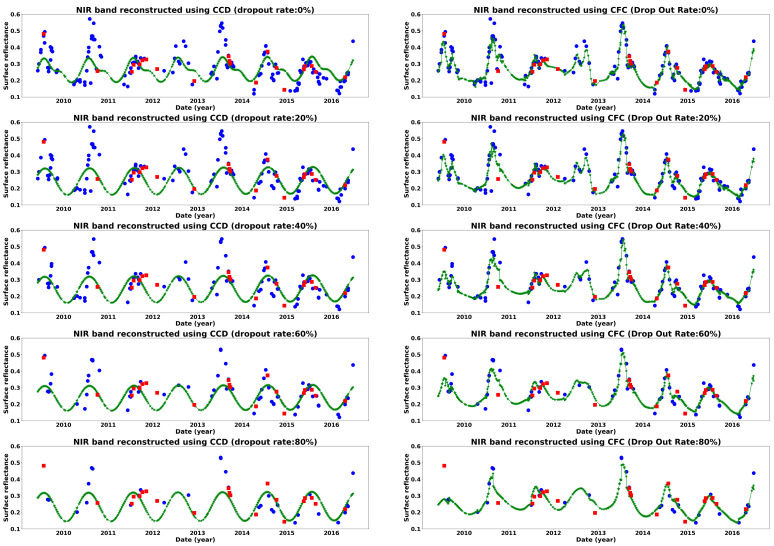

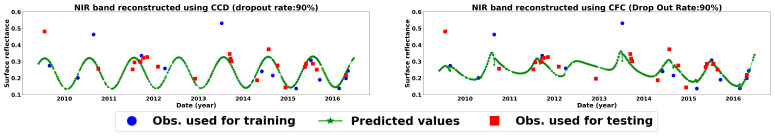
The effect of density level on reconstructing a sample cropland time series using CCD (**left**) and CFC (**right**).

Figure 17 illustrates the effect of observation density on reconstructing the NIR band for a sample cropland pixel as an example. In the case of CCD, consistent with the findings shown in Figure 14, Figure 15 and Figure 16, decreasing observation density has minimal impact on the Landsat time series reconstruction. Notably, after a 20% reduction in observations, CCD employs a simpler harmonic model to represent the time series, which remains more stable with lower-density observations. In contrast, CFC exhibits greater variation in response to changes in observation density, highlighting its greater dependence on observation density. Additionally, at high observation densities, CFC shows signs of overfitting, indicating its susceptibility to noise within the time series data. Applying noise reduction or increasing regularization in CFC may help mitigate this issue and improve CFC’s robustness to noise.

In a further analysis, we investigated the effect of observation density on reconstructing two adjacent segments of the Landsat time series, each spanning seven years but with different satellite coverage. The first period, from 1992 to 1999, represents a single-satellite scenario where only Landsat 5 was operational. The second period, from 2000 to 2007, represents a double-satellite scenario, with both Landsat 5 and Landsat 7 actively capturing imagery.

**Figure 18:**
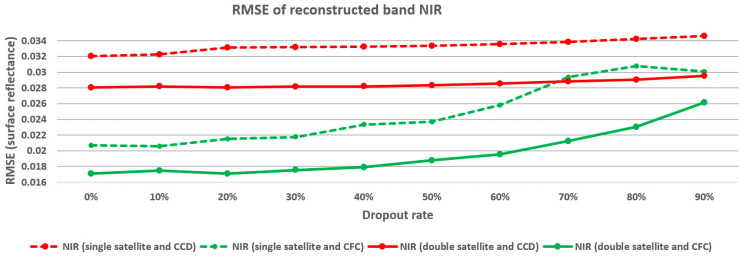
Relation between observation density and accuracy of NIR band reconstruction for different parts of Landsat time series with different numbers of active satellites.

Figure 18 presents the results of reconstructing the NIR band at different observation densities for the single-satellite case (14 test images) and the dual-satellite case (30 test images) using CFC and CCD methods. The results show that the CFC method achieved higher reconstruction accuracy across all density levels. Additionally, in the dual-satellite period, both CFC and CCD demonstrated improved reconstruction performance, in large part due to the increased frequency of observations provided by two active satellites. These findings highlight that denser time series data, enabled by multiple satellites, significantly enhance the reconstruction quality. The dual-satellite period allows for more consistent data capture, which benefits both methods, particularly the CFC method, which relies heavily on observation density. This improvement underscores the potential advantage of multi-satellite coverage in achieving reliable reconstructions, especially in regions or periods with sparse observational data.

To summarize the findings of this research, some of the key points are outlined in the following section. CFC has been shown here to be superior for estimating missing observations compared to CCD and thus has potential to enhance many RS time series applications. The results also indicated that the predicted images using CFC were much closer to real observed images compared to CCD. Even at low density (around 2 observations per year), the CFC results were more accurate, and the shape of the time series was maintained relative to the full time series results. Only at very low density did CFC deviate such that its use for applications was considered compromised. For example, considering time series reconstruction at a 90% dropout rate (Figure 17), the CFC results have much more strongly deviated from the full time series results compared to CCD. Thus, CFC is applicable where there are a sufficient number of observations, which, based on these results, is an average of four observations per year (the case of an 80% dropout rate). Clearly, where these observations occur in the time series is important, as shown by [38].

The density of clear-sky observations in optical RS data varies globally due to factors beyond the satellite’s revisit cycle [39]. Regions near the equator, with limited scene overlap, northern areas experiencing rapid seasonal changes, and locations with frequent cloud cover such as coastal and mountainous zones present significant challenges for time series modeling [7]. Given satellite revisit limitations, the early Landsat archive from 1985 to 1999 is anticipated to be more problematic in these areas. However, higher-density time series data such as those from MODIS and Sentinel 2 are expected to yield more accurate results.

For time series classification, a potential advantage of CCD is that it provides between 4 and 8 model parameters indicating the structure of the time series [37]. However, with deep learning methods, this is not a feasible approach as there are a huge number of parameters. For example, the CFC model (CFC-mmRNN) used in this study includes 202,374 parameters. However, with better estimation of the time series using CFC, derived spectral–temporal metrics should be better characterized and theoretically provide enhanced input for classification.

In the examples presented in this study, croplands exhibited greater temporal complexity than grasslands, leading to lower accuracies in time series reconstruction. However, CFC provided greater improvements over CCD for cropland pixels, which have more complex patterns, and smaller improvements for grassland pixels, with less complex patterns. This is also an important consideration as the extent of CFC’s improvement over CCD can vary depending on the land cover type, density level, spectral bands, or indices analyzed. The results for winter showed the lowest accuracy among other seasons. Snow was removed from the time series using Fmask and Tmask, significantly reducing the number of clear observations available during this season. Additionally, the snow masking used in the study is not entirely accurate, and this results in higher reflectance variability due to snow compared to other seasons. These factors contributed to the similar performance observed between CFC and CCD in winter for some spectral bands.

## 4. Conclusions

The study demonstrated the capabilities of the CFC-based deep neural network in reconstructing historical Landsat time series data, significantly advancing beyond the performance of the commonly used CCD method. Specifically, CFC improved reconstruction accuracy by 33% to 42% across the six bands analyzed, demonstrating superior capability in handling irregular and sparse time series data. The CFC method consistently achieved higher reconstruction accuracy across all spectral bands and seasons, except for the SWIR bands in winter, where its performance was similar to the CCD method due to the very sparse time series during the winter months.

One of the notable strengths of the CFC method is its ability to maintain the integrity of time series shapes, even at lower observation densities. The study revealed that CFC provided accurate reconstructions, closely matching the observed images, particularly for regions with complex temporal patterns like croplands. In test image reconstructions, CFC produced narrower confidence intervals and was able to predict missing values in the time series with significantly higher precision than CCD. Furthermore, the CFC method’s robustness in handling varying observation densities was evident. Although both CFC and CCD showed reduced performance with fewer clear observations, CFC consistently outperformed CCD at all density levels. This highlights CFC’s potential for enhancing RS applications, particularly in areas where data are sparse or irregular. The analysis also underscored the benefits of multi-satellite coverage. During periods with dual-satellite observations, both CFC and CCD demonstrated improved reconstruction performance due to increased data density.

CFC offers a promising solution for reconstructing historical Landsat data, enhancing the quality and reliability of time series analysis. Future research should focus on optimizing CFC’s robustness against noise and exploring its applicability to other datasets, such as MODIS and Sentinel-2. Integrating CFC with noise reduction techniques or adding regularization methods could further improve its performance, paving the way for more accurate environmental monitoring and assessment. By addressing these challenges, the CFC method can significantly contribute to the field of RS, providing a robust framework for analyzing complex and irregular time series data and supporting various environmental applications.

## Figures and Tables

**Table 1:** The number of test images selected randomly from the image set with at least 60% clear observations and the number of training images with less and more than 60% clear observations.

Season	Spring	Summer	Fall	Winter
Months	March, April May	June, July, August	September, October, November	December, January, February
Number of test images	25	49	35	6
Number of training images with more than 60% of clear observation	179	328	234	40
Number of training images with less than 60% of clear observation	98	84	49	38

**Table 2:** Results of test image reconstruction based on RMSE.

Band	RMSE of Image Reconstruction Using CCD	RMSE of Image Reconstruction Using CFC	Improvement of CFC Over CCD
Blue	0.01492	0.00983	34%
Green	0.01876	0.01228	35%
Red	0.0255	0.01488	42%
NIR	0.03201	0.01978	38%
SWIR1	0.03928	0.02574	34%
SWIR2	0.03927	0.02262	42%

## Data Availability

The Landsat data used in the study are openly available in Google Earth Engine at https://developers.google.com/earth-engine/datasets/catalog/landsat (accessed on 23 July 2024).

## References

[1] Wulder M.A., Roy D.P., Radeloff V.C., Loveland T.R., Anderson M.C., Johnson D.M., Healey S., Zhu Z., Scambos T.A., Pahlevan N. (2022). Fifty years of Landsat science and impacts. Remote Sens. Environ..

[2] Hemati M., Hasanlou M., Mahdianpari M., Mohammadimanesh F. (2021). A systematic review of landsat data for change detection applications: 50 years of monitoring the earth. Remote Sens..

[3] Wulder M.A., Loveland T.R., Roy D.P., Crawford C.J., Masek J.G., Woodcock C.E., Allen R.G., Anderson M.C., Belward A.S., Cohen W.B. (2019). Current status of Landsat program, science, and applications. Remote Sens. Environ..

[4] Zhu Z., Wulder M.A., Roy D.P., Woodcock C.E., Hansen M.C., Radeloff V.C., Healey S.P., Schaaf C., Hostert P., Strobl P. (2019). Benefits of the free and open Landsat data policy. Remote Sens. Environ..

[5] Zhu Z., Woodcock C.E. (2014). Continuous change detection and classification of land cover using all available Landsat data. Remote Sens. Environ..

[6] Frantz D., Röder A., Udelhoven T., Schmidt M. (2015). Enhancing the detectability of clouds and their shadows in multitemporal dryland Landsat imagery: Extending Fmask. IEEE Geosci. Remote Sens. Lett..

[7] Pouliot D., Latifovic R. (2018). Reconstruction of Landsat time series in the presence of irregular and sparse observations: Development and assessment in north-eastern Alberta, Canada. Remote Sens. Environ..

[8] Ataabadi M.B., Pouliot D., Chen D., Oluwadare T.S. Reconstructing Missing Data in Historical Landsat Images Using Advanced Deep Learning Algorithms. Proceedings of the Asia-Pacific Remote Sensing.

[9] Whitney K., Scudiero E., El-Askary H.M., Skaggs T.H., Allali M., Corwin D.L. (2018). Validating the use of MODIS time series for salinity assessment over agricultural soils in California, USA. Ecol. Indic..

[10] Qiu S., Zhu Z., He B. (2019). Fmask 4.0: Improved cloud and cloud shadow detection in Landsats 4–8 and Sentinel-2 imagery. Remote Sens. Environ..

[11] Tang Z., Amatulli G., Pellikka P.K., Heiskanen J. (2021). Spectral Temporal Information for Missing Data Reconstruction (STIMDR) of Landsat Reflectance Time Series. Remote Sens..

[12] Zhang J., Shang R., Rittenhouse C., Witharana C., Zhu Z. (2021). Evaluating the impacts of models, data density and irregularity on reconstructing and forecasting dense Landsat time series. Sci. Remote Sens..

[13] Zhu Z., Woodcock C.E., Holden C., Yang Z. (2015). Generating synthetic Landsat images based on all available Landsat data: Predicting Landsat surface reflectance at any given time. Remote Sens. Environ..

[14] Zhou Q., Zhu Z., Xian G., Li C. (2022). A novel regression method for harmonic analysis of time series. ISPRS J. Photogramm. Remote Sens..

[15] Tollerud H.J., Zhu Z., Smith K., Wellington D.F., Hussain R.A., Viola D. (2023). Toward consistent change detection across irregular remote sensing time series observations. Remote Sens. Environ..

[16] Zhou Y.n., Wang S., Wu T., Feng L., Wu W., Luo J., Zhang X., Yan N. (2022). For-backward LSTM-based missing data reconstruction for time-series Landsat images. GIScience Remote Sens..

[17] Weerakody P.B., Wong K.W., Wang G., Ela W. (2021). A review of irregular time series data handling with gated recurrent neural networks. Neurocomputing.

[18] Zhang H.K., Luo D., Li Z. (2024). Classifying raw irregular time series (CRIT) for large area land cover mapping by adapting transformer model. Sci. Remote Sens..

[19] Baytas I.M., Xiao C., Zhang X., Wang F., Jain A.K., Zhou J. Patient subtyping via time-aware LSTM networks. Proceedings of the 23rd ACM SIGKDD International Conference on Knowledge Discovery and Data Mining.

[20] Chen R.T., Rubanova Y., Bettencourt J., Duvenaud D.K. (2018). Neural ordinary differential equations. Adv. Neural Inf. Process. Syst..

[21] Prince P.J., Dormand J.R. (1981). High order embedded Runge-Kutta formulae. J. Comput. Appl. Math..

[22] Hasani R., Lechner M., Amini A., Liebenwein L., Ray A., Tschaikowski M., Teschl G., Rus D. (2022). Closed-form continuous-time neural networks. Nat. Mach. Intell..

[23] Encabo J.B.M., Cordeiro M.R., Badreldin N., McGeough E.J., Walker D. (2023). Assessment of remotely sensed inventories for land cover classification of public grasslands in Manitoba, Canada. Grass Forage Sci..

[24] Olimb S.K., Dixon A.P., Dolfi E., Engstrom R., Anderson K. (2018). Prairie or planted? Using time-series NDVI to determine grassland characteristics in Montana. GeoJournal.

[25] McInnes W.S., Smith B., McDermid G.J. (2015). Discriminating native and nonnative grasses in the dry mixedgrass prairie with MODIS NDVI time series. IEEE J. Sel. Top. Appl. Earth Obs. Remote Sens..

[26] Lindsay E.J., King D.J., Davidson A.M., Daneshfar B. (2019). Canadian prairie rangeland and seeded forage classification using multiseason Landsat 8 and Summer RADARSAT-2. Rangel. Ecol. Manag..

[27] Rubanova Y., Chen R.T., Duvenaud D.K. (2019). Latent ordinary differential equations for irregularly-sampled time series. Adv. Neural Inf. Process. Syst..

[28] Lechner M., Hasani R. (2020). Learning long-term dependencies in irregularly-sampled time series. arXiv.

[29] Paoletti M.E., Haut J.M., Plaza J., Plaza A. (2019). Neural ordinary differential equations for hyperspectral image classification. IEEE Trans. Geosci. Remote Sens..

[30] Ayed I., de Bézenac E., Pajot A., Gallinari P. (2022). Modelling spatiotemporal dynamics from Earth observation data with neural differential equations. Mach. Learn..

[31] Qiu W., Zhang W., Wang G., Guo Z., Zhao J., Ma K. (2024). Combined wind speed forecasting model based on secondary decomposition and quantile regression closed-form continuous-time neural network. Int. J. Green Energy.

[32] Raissi M., Perdikaris P., Karniadakis G.E. (2019). Physics-informed neural networks: A deep learning framework for solving forward and inverse problems involving nonlinear partial differential equations. J. Comput. Phys..

[33] Zhu Z., Woodcock C.E. (2012). Object-based cloud and cloud shadow detection in Landsat imagery. Remote Sens. Environ..

[34] USGS Landsat 7. https://www.usgs.gov/landsat-missions/landsat-7.

[35] Qiu S., Zhu Z., Shang R., Crawford C. (2021). Can Landsat 7 preserve its science capability with a drifting orbit?. Sci. Remote Sens..

[36] Kingma D.P. (2014). Adam: A method for stochastic optimization. arXiv.

[37] Xian G.Z., Smith K., Wellington D., Horton J., Zhou Q., Li C., Auch R., Brown J.F., Zhu Z., Reker R.R. (2022). Implementation of the CCDC algorithm to produce the LCMAP Collection 1.0 annual land surface change product. Earth Syst. Sci. Data.

[38] Frantz D., Rufin P., Janz A., Ernst S., Pflugmacher D., Schug F., Hostert P. (2023). Understanding the robustness of spectral-temporal metrics across the global Landsat archive from 1984 to 2019–a quantitative evaluation. Remote Sens. Environ..

[39] Kovalskyy V., Roy D.P. (2013). The global availability of Landsat 5 TM and Landsat 7 ETM+ land surface observations and implications for global 30 m Landsat data product generation. Remote Sens. Environ..

